# Fullerenes for the treatment of cancer: an emerging tool

**DOI:** 10.1007/s11356-022-21449-7

**Published:** 2022-07-06

**Authors:** Neha Benedicta Fernandes, Raghavendra Udaya Kumar Shenoy, Mandira Kashi Kajampady, Cleona E. M. DCruz, Rupesh K. Shirodkar, Lalit Kumar, Ruchi Verma

**Affiliations:** 1 Department of Pharmaceutics, Manipal College of Pharmaceutical Sciences, Manipal Academy of Higher Education, Manipal, 576104 Udupi, Karnataka India; 2 Department of Pharmaceutics, Goa College of Pharmacy, 18th June Road, Panaji, 403 001 Goa India; 3 Department of Pharmaceutical Chemistry, Manipal College of Pharmaceutical Sciences, Manipal Academy of Higher Education, Manipal, 576104 Udupi, Karnataka India

**Keywords:** Nanotechnology, Biosensing, Cancer therapy, Photodynamic therapy, Biocompatibility, Biodegradability, Nanomedicine

## Abstract

Cancer is a most common cause of mortality globally. Available medicines possess severe side effects owing to their non-specific targeting. Hence, there is a need of an alternative in the healthcare system that should have high efficacy with the least side effects, also having the ability to achieve site-specific targeting and be reproducible. This is possible with the help of fullerenes. Fullerenes are having the unique physicochemical and photosensitizer properties. This article discusses the synthesis, functionalization, mechanism, various properties, and applications of C60 fullerenes in the treatment of cancer. The review article also addresses the various factors influencing the activity of fullerenes including the environmental conditions, toxicity profile, and future prospective.

**Graphical abstract:**

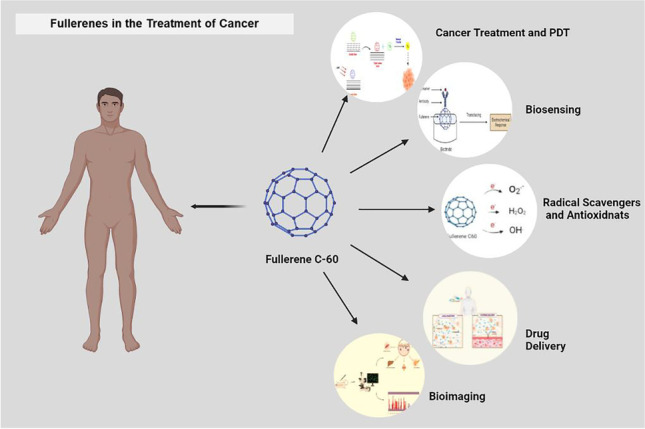

## Introduction

For a few decades, nanoparticles (NPs) have gained the limelight in the treatment of cancer over a combination of conventional cancer therapies such as chemotherapy, surgery, radiation therapy, immunotherapy, and hormone therapy (Jabir et al. 2018). Chemotherapeutics pose pharmaceutical restraints, which include problems with physicochemical stability and aqueous solubility. They also exhibit dose-limiting toxicity and non-specific toxicity to healthy cells, with alopecia, anorexia, peripheral neuropathy, and diarrhea being the distinctive side effects. Another notable challenge impeding cancer treatment is multidrug resistance (MDR) whereby the cancer cells become cross-resistant to several anti-neoplastic agents used. Nanotechnology presents a cutting-edge treatment for cancer with its decreased systemic toxicity by targeting the specific tissues with their functionalization. This technique also provides an unusual strategy to evade multidrug resistance as they can bypass the drug efflux mechanism. In addition to the merits, they also propose the treatment sphere, and nanoparticles have also emerged as diagnostic assets (Awasthi et al. 2018).

Numerous classes of nanotechnology-based products such as liposomes, dendrimers, colloidal silica NPs, magnetic nanoparticles, polymeric micelles, and solid-lipid nanoparticles (SLNs) are some nanocarriers that have been used in cancer therapy. The currently developed nanomedicine and nanodevices such as quantum dots, nanowires, nanotubes, nano cantilevers, nanopores, nano-shells, and nanoparticles have also been deemed promising for numerous cancer treatments. The carbon-based nanomaterials possess notable intrinsic properties including the assortment of carbon nanotubes (CNTs), graphene oxide (GO), nano diamonds, graphene quantum dots (GQDs), and fullerenes (Li et al. 2015b; Awasthi et al. 2018).

Carbon nanomaterials (CNMs) hold exceptional physicochemical properties, including thermal, optical, electrical, mechanical, and structural diversity, compared to other nanoparticles (Hong et al. 2015). These excellent characteristics possessed by the hollow cylindrical graphitic sheets, the carbon nanotubes (CNTs), give them greater flexibility, strength, and electrical conductivity towards biological entities, which is beneficial for the medical diagnosis and treatment (Wu et al. 2010; Hwang et al. 2013; Roldo and Fatouros 2013; Shanbhag and Prasad 2016). Contrarily, the planar graphitic sheets, the graphenes, can be easily surface-modified using various functional groups, which then aids in the specific and selective detection of numerous biological moieties. Additionally, its vast surface area, chemical purity, and free *π*-electrons provide it an ultimate candidate for the delivery of drugs (Yang et al. 2013a; Zhang et al. 2013; Pattnaik et al. 2016; Wang et al. 2016). Its achievable performance towards distinct fluorescent dyes, APIs, and other biomaterials makes it an extensively useful tool in vivo imaging, diagnosis, and cancer treatment.

GQDs possess comparatively exceptional biocompatibility and resistance to photo-bleaching than other fluorescent dyes or semiconductor quantum dots and provide superior photoluminescence due to quantum confinement. The GQDs endowed with superior graphene-like properties of large surface area and available *π* electrons make them an innovative nanomaterial for imaging, targeted drug delivery, biomolecular sensing, cancer therapy, and other biomedical applications (Zheng et al. 2015; Chen et al. 2017; Kumawat et al. 2017; Li et al. 2017). The most prevalent fullerene, C_60_, is a stable icosahedron with C_5_–C_5_ single bonds forming 12 pentagons and C_5_–C_6_ double bonds forming 20 hexagons (Krätschmer et al. 1990). It consists of 30 double bonds that readily accept free radicals, hence, giving it the term “free radicle sponge” (Krusic et al. 1991). This singular characteristic of C_60_ to either quench or generate cell-damaging reactive oxygen species (ROS), as well as its small size and large surface area, could be efficiently applied in biomedicine and clinical therapy (Markovic and Trajkovic 2008).

The two-fold property of reactive free radical quenching in combination with acting as targeted drug carriers of the fullerenes makes the formulation therapeutically more effective. Polyhydroxy fullerenes (PHFs) possess excellent aqueous-solubility, biocompatibility, and biodegradability and exhibit superior antioxidant properties apart from preventing allergic responses and protecting CNS tissues as compared to gold-based nanoparticles and carbon nanotubes. Moreover, PHF shows distinctive tumor growth inhibition and promotes immune system upregulation. PHF molecules have a size of 1.3 nm which can be easily secreted in urine, whereas larger nanocarriers such as carbon nanotubes and gold-based nanoparticles typically surpass the renal excretion limit of 5.5 nm (Krishna et al. 2010).

Functionalized fullerenes possess characteristics that support anti-cancer therapy apart from tumor inhibition and help combat systemic toxicity and drug resistance which are commonly encountered in the conventional chemotherapeutic approach (Liu et al. 2009b; Li et al. 2011). PDT is a broadly accepted valuable treatment preference for malignant and non-neoplastic diseases and currently, along with the progress in fiber-optic systems, light can be supplied precisely to various body parts for the treatment of tumors. Thus, photodynamic therapy (PDT) applications have expanded to numerous endoscopically available tumors, including lung, gastric, cervical, bladder, head, and neck cancers. PDT due to its advantages holds an upper hand over chemotherapy and radiotherapy. It demonstrates no long-term side effects when an efficient photosensitizer is employed. The procedure is minimally invasive in comparison to surgery and results in barely any scarring. It has the ability to deliver extremely targeted therapy at the disease site and allows repeatable treatments at the same location if necessary. PDT is less expensive than other cancer treatments and since it can be completed in a short time, this allows treatment as an outpatient (Huang et al. 2014).

In 2005, Chen et al. showed that gadolinium endohedral metallofullerenes (Gd@C_82_(OH)n nanoparticles) could effectively inhibit the growth of murine H22 hepatoma without adversely affecting vital organs (Chen et al. 2005), exhibiting an edge over conventional antitumor drugs. Zhu et al. also verified the tumor-inhibitory effect of C_60_(OH)*x* on the same model and observed substantial tumor inhibition rates and decreased liver damage (Zhu et al. 2008). Moreover, fullerenol C_60_(OH)_20_ showed anti-metastatic activity in cancer metastasis models (Jiao et al. 2010). It was observed that certain fullerenes could enhance the chemo-sensitization of tumor cells to chemotherapeutic agents in the case of drug-resistant cancer cells (Zhang et al. 2009; Liang et al. 2010). Conjugating endohedral metallofullerenes, e.g., gadolinium metallofullerenes, with molecules like fluorescent proteins and interleukins having receptors expressed on tumors could aid dually in imaging and tumor targeting (Shu et al. 2008; Fillmore et al. 2011). The use of acoustic-explosive mechanisms and photothermal ablation with certain functionalized fullerenes, like carboxy fullerenes (CF) and polyhydroxy fullerenes (PHF), shows rapid tumor shrinkage on near-infrared irradiation (Krishna et al. 2010).

## Properties of fullerenes

The unique structural features of fullerenes have ratified them in various fields. They have a soccer ball–like spherical shape made up of 60 carbon atoms, 12 pentagons with C_5_–C_5_ single bonds, and 20 hexagons with C_5_–C_6_ double bonds (Kroto et al. 1985). They are comprised of fused rings and conjugated bonds with a hybridization of sp^2^ and sp^3^ bonds, with the average bond length of the single bond, which is 0.145 nm and the double bond that is 0.141 nm (Zhang et al. 1991).

They bear a truncated icosahedral symmetry because of which every carbon atom environment remains identical (Zhou and Wilson 2005). Fullerene C_60_ has the smallest cage structure which makes it highly reactive. It is also highly stable as it follows the isolated pentagon rule (Bingel and Schiffer 1995) which states that all the pentagons are surrounded by 5 hexagons (Manolopoulos and Fowler 1992). They are electron deficient, because of poor electron delocalization, which makes them a potent antioxidant and is widely used in cancer therapy (Prato 1997; Echegoyen and Echegoyen 1998). Fullerenes are extremely hydrophobic in nature because of which their solubility is low in polar solvents and high in organic solvents such as benzene, toluene, chloroform (Kadish and Ruoff 2000). To increase its solubility in polar solvents, the derivatization of fullerenes with polar groups has been carried out. They can undergo a lot of chemical reactions because they are electron acceptors. Due to its inertness, a lot of ionic species can be enclosed in its cage-like structure. The several distinct properties of high hydrophobicity, high cohesivity between fullerene molecules, photoactivity, high reactivity, and ability to accept and release electrons allow varied chemical transformations and structural modifications for extensive biomedical use (Table 1).

**Table 1:** Physical properties of fullerene (Yadav 2018)

Sr. no	Properties	Value
1	Density	1.65 g/cm^3^
2	Refractive index	2.2 (600 nm)
3	Melting point	260 °K
4	Boiling point	Sublimes at 800 °K
5	Standard heat of formation	9.08 K Cal/mol
6	Resistivity	1014 Ohms/m
7	Vapor pressure	5 × 10^−6^ torr at room temperature
8	Thermal conductivity	0.4 W/mK
9	Young’s modulus	14 GPa

## Mechanism of action of fullerenes in tumor therapy

### Antioxidant

Normal cellular processes and some abnormal reactions in mitochondria lead to the generation of free radicals. The excess production of these ROS-like peroxides, hydroxyl radicals, superoxide radicals, and singlet oxygen can cause cellular damage and eventually lead to cancer (Dugan et al. 1996; Wang et al. 2006; Markovic and Trajkovic 2008; Yin et al. 2009). The intrinsic free radical scavenging property of fullerene is a breakthrough in the treatment of cancer. Because of the small size, fullerenes show enhanced permeability and retention effect (EPR) in the tumor mass, decreasing the ROS concentration without being consumed and thus acts as a free radical sponge (Singh and Lillard 2009). It also inhibits the activation of proto-oncogenes, tumor growth, and angiogenesis fostering in anticancer activity (Chen et al. 2012).

Since fullerenes are electron-deficient in nature owing to the poor delocalization of electrons, they are highly reactive towards the free radicals. The *π*–*π* carbon bonds in the fullerene structure abstract of the electrons thus making it behave as an antioxidant (Krusic et al. 1991; Echegoyen and Echegoyen 1998).

The radical scavenging activity of fullerenes can also occur when there is reduction and re-oxidation between the fullerenes and ROS by exchanging the electrons present on the outer shell. The surface of the fullerene reacts with ROS, followed by addition or elimination steps, ultimately leading to the catalytic cleavage of ROS. These two distinct antioxidant mechanisms have been considered while developing fullerenes in cancer therapy. The fullerenes being hydrophobic in nature allow enhancement in antioxidant property by derivatizing them with polar groups (Kwag et al. 2012).

### Photodynamic therapy

**Fig. 1:**
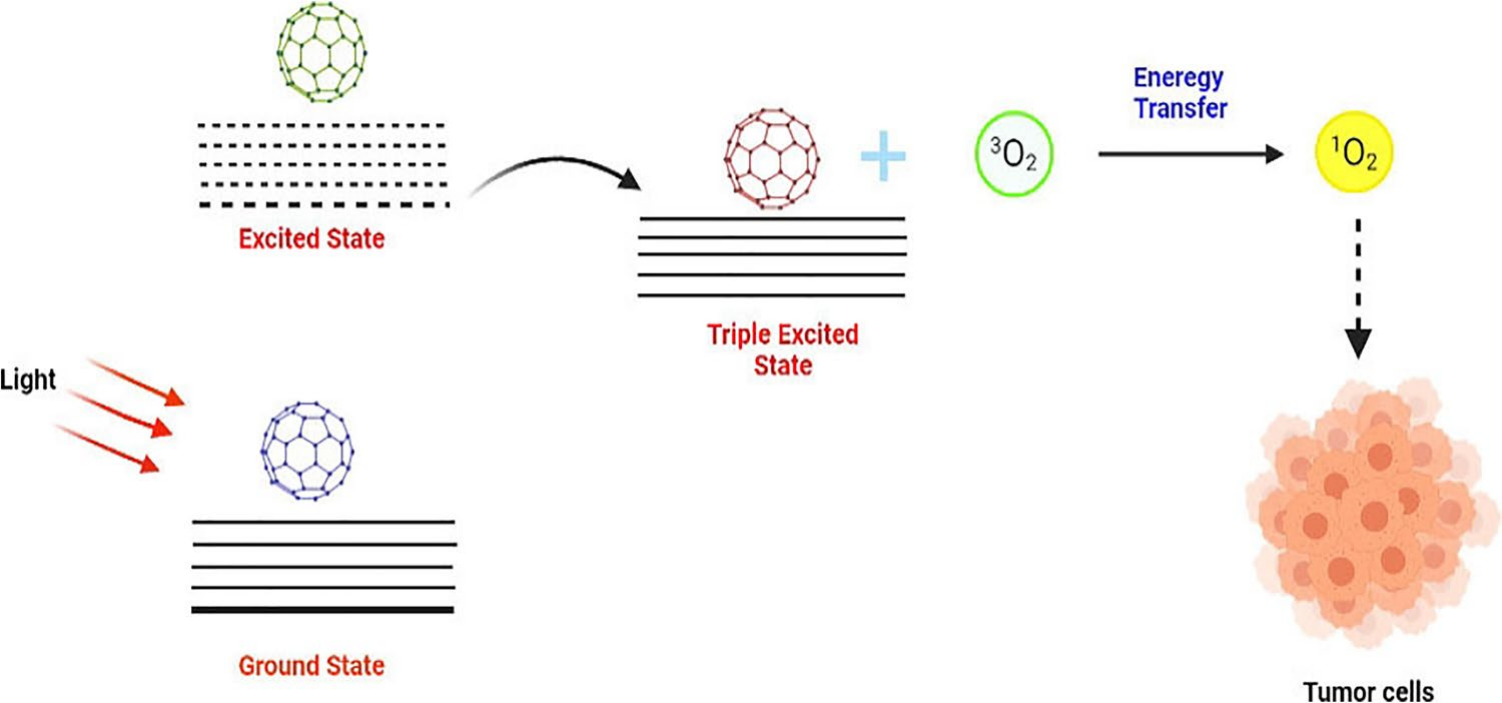
Fullerene as a photosensitizer in photodynamic therapy

Cancer therapy by fullerenes can also be achieved by photodynamic therapy (PDT) (schematic shown in Fig. 1). It is a non-invasive treatment that involves the generation of reactive oxygen species in a localized area, leading to the destruction of cancer cells through various pathways and thus showing targeted drug delivery (Jiang and Li 2007; Kwag et al. 2012). The fullerenes undergo photoexcitation upon illumination, absorb radiation of a particular wavelength, and get excited from the ground state (S_0_) ^1^C_60_ to an excited state (S_1_) which is short-lived (Broekgaarden et al. 2015). It then undergoes non-radiative decay to a lower lying state called the triplet state (T_1_) ^3^C_60_ which is long-lived by a process called inter-system crossing (Sharma et al. 2011). In the presence of molecular oxygen, while moving from triplet to ground state, the fullerene transfers its energy to O_2_, generating a singlet oxygen ^1^O_2_, which has been observed to be a highly effective cytotoxic species (Yamakoshi et al. 2003).

For its anti-cancer properties, free radicals from fullerenes can be generated by two major mechanisms. In type 1 photochemical mechanism, common reducing agents like NADH or NADPH present in the cell donate electrons to the fullerenes, which makes it possess an additional unpaired electron behaving like a radical anion C_60_^−^. When this reacts with oxygen, it transfers an electron to produce a reactive oxygen species like superoxide or hydroxyl radical. (Yamokshi et al. 2003; Mroz et al. 2008).

The type 2 reaction involves the direct transfer of energy to the molecular oxygen to generate a singlet oxygen which is highly cytotoxic in nature.

## Synthesis of fullerenes

### Arc discharge method

One of the oldest methods of fullerene production by the vaporization and condensation of a graphite rod was developed in Germany by Kratschmer and Huffman in 1990 (Krätschmer et al. 1990). Although its production is large but costs economical, it has continued to remain a popular and well-established process (Churilov 2008).

**Fig. 2:**
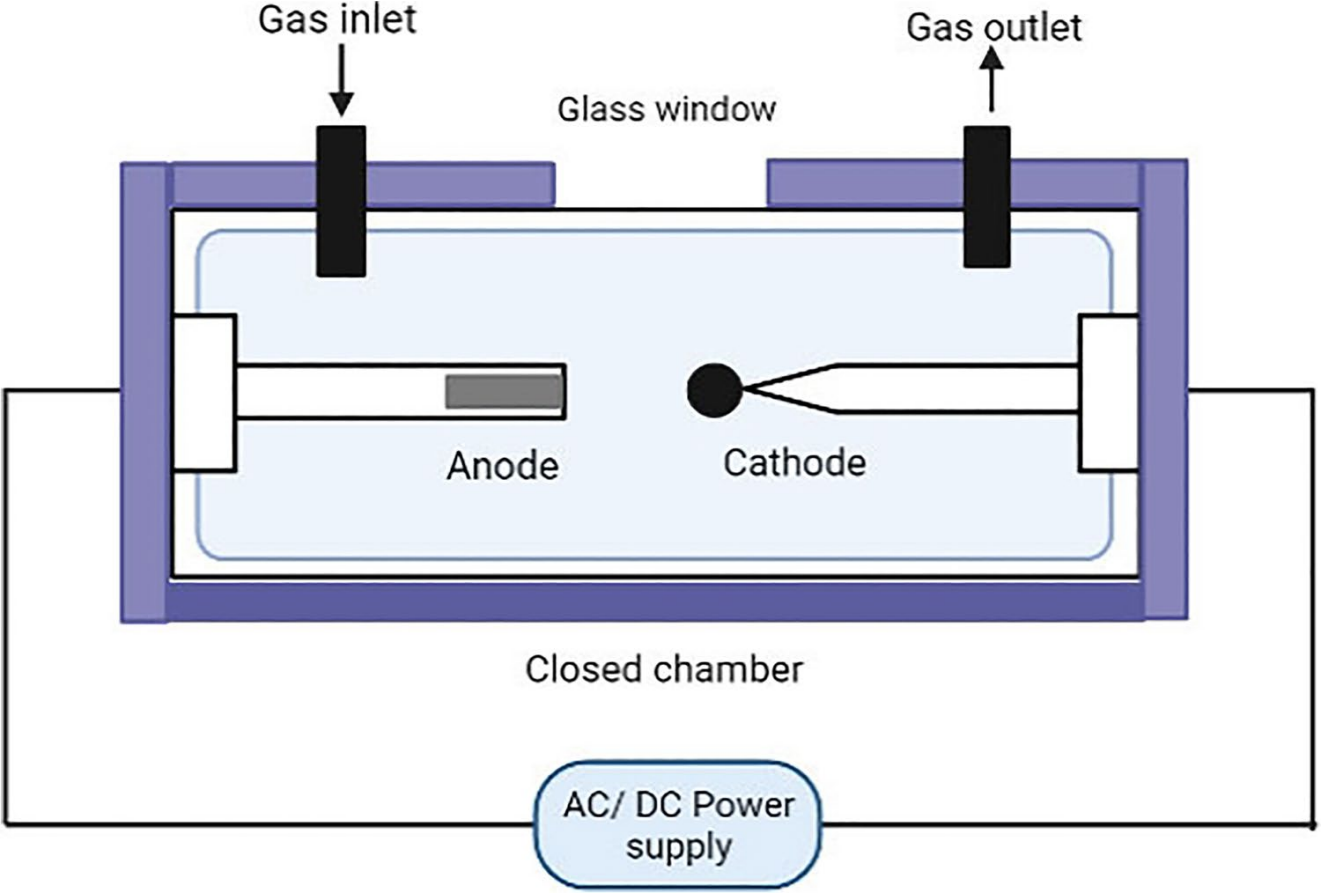
Arc discharge method

The method involves vaporizing graphite rods which are the carbon source by direct or alternative electric current in a neutralized environment which is provided by inert gases like helium and argon (Churilov 2008; Hare et al. 2013). At high temperatures, the electric discharge between the anode and the cathode graphite rods creates plasma. The most frequently used apparatus in the arc discharge method (schematic shown in Fig. 2) is the tube-shaped reactor which is made of stainless steel to maintain its rigidity and strength when subjected to external air pressure. It consists of a furnace, two graphite electrodes, two water inlets, and two water outlets for bell jars which are accessible from outside the reactor, an inert gas inlet, a vacuum pump outlet, and a plasma observation window along with an AC/DC power supply source connected to each electrode end from outside the reactor (Brinkmann and Dress 1997; Bezmelnitsin et al. 1997; Sugai et al. 2000).

The tube-shaped reactor is filled with inert gas whose pressure is maintained at 100 Torr. The temperature of the reactor is between 500 and 1000 °C, and a current of 70 A and voltage of 20 V are applied between the anode and cathode graphite rods which are placed few millimeters apart. The arc discharge between the graphite electrodes produces plasma that vaporizes the graphite rods. At this point, the temperature of the graphite rods increases which is then cooled using the water in glass bell jars around each rod. Because of the condensation process, fullerene soot is produced during the process which is collected using a vacuum pump outlet. The fullerene extraction or purification of the fullerene soot can either be carried out using a Soxhlet extractor that produces large quantities of fullerenes (Khemani et al. 1992) or by a more traditional method, i.e., washing the soot with hydrocarbons like hexane (Jehlička et al. 2005).

### Laser ablation method

In this method, the carbon source is ablated by a laser in the presence of an inert gas (argon) at a high temperature (Mordkovich et al. 2002). The apparatus for laser ablation consists of a tube-shaped glass, an optical pyrometer to measure the temperature of the graphite rod, a vacuum pump outlet, and a motor which is used to rotate the graphite rod, an argon gas inlet, and an argon gas flow meter. Two focus lenses are placed perpendicularly to the laser beams along with a laser energy meter and two laser generators which produce fundamental (1064 nm), second (532 nm), and third-harmonic (355 nm) light with a repetition of 10 Hz (Dietz et al. 1981).

The graphite rod which provides the carbon source is placed at the center of the reactor and is connected to a motor which rotates it (Kasuya et al. 2002; Azami et al. 2008).

**Fig. 3:**
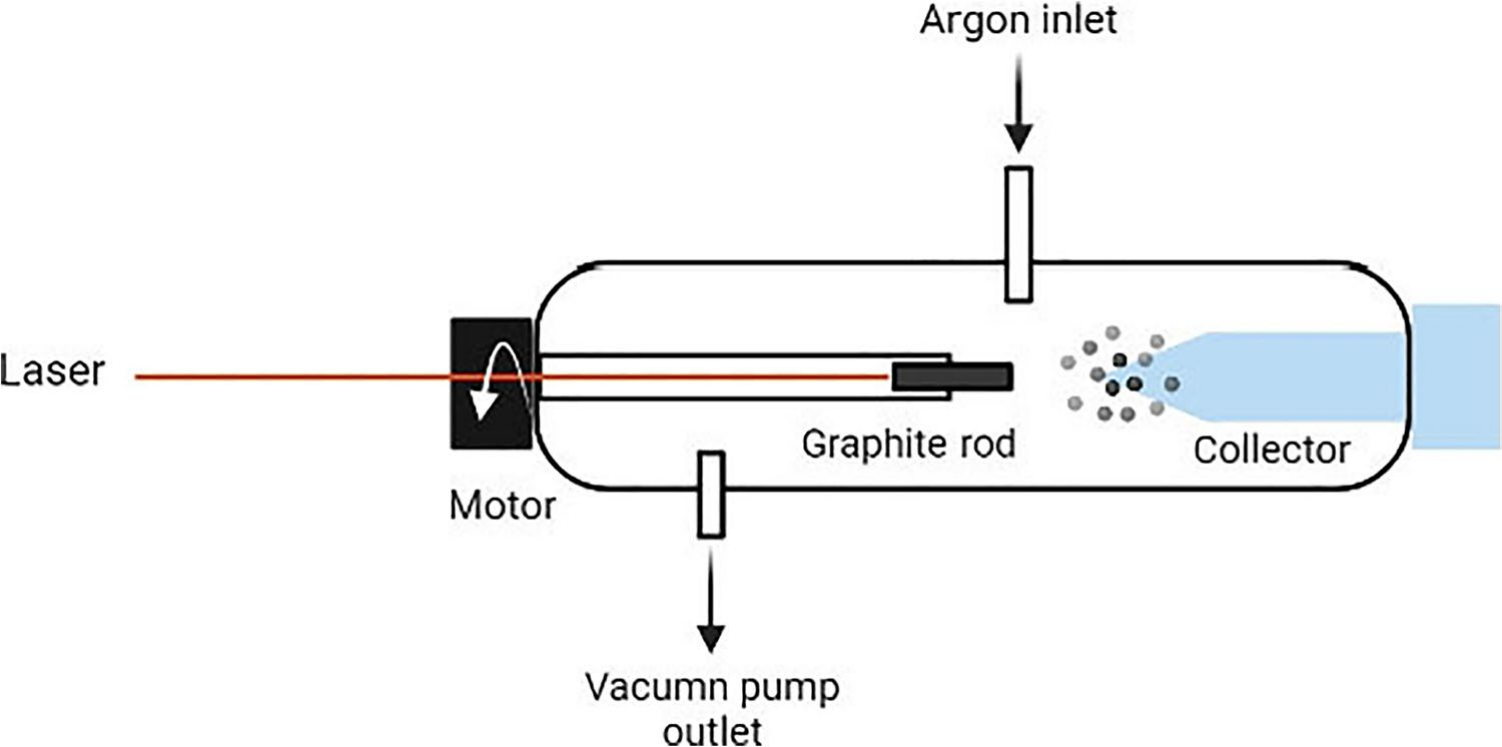
Laser ablation method

Argon is then pumped into the reactor (pressure range of 53–80 kPa) to provide a neutralized (neutralized) environment. The laser ablation of the graphite rod begins as it rotates at the rate of 20 rpm. After the graphite rod has been completely ablated, the soot present in the reactor tube can be extracted using a Soxhlet extractor (schematic shown in Fig. 3).

### Combustion method

This method involves the combustion of hydrocarbons in the presence of oxygen and argon to produce fullerenes. The hydrocarbons or a premixed benzene/oxygen fuel provide the carbon source for the formation of soot. The apparatus is made of a stainless steel, tube-shaped generator from which air is removed. The chamber consists of a fuel injection system that injects the premixed fuel of benzene/oxygen/argon that would be ignited by a burner platform.

**Fig. 4:**
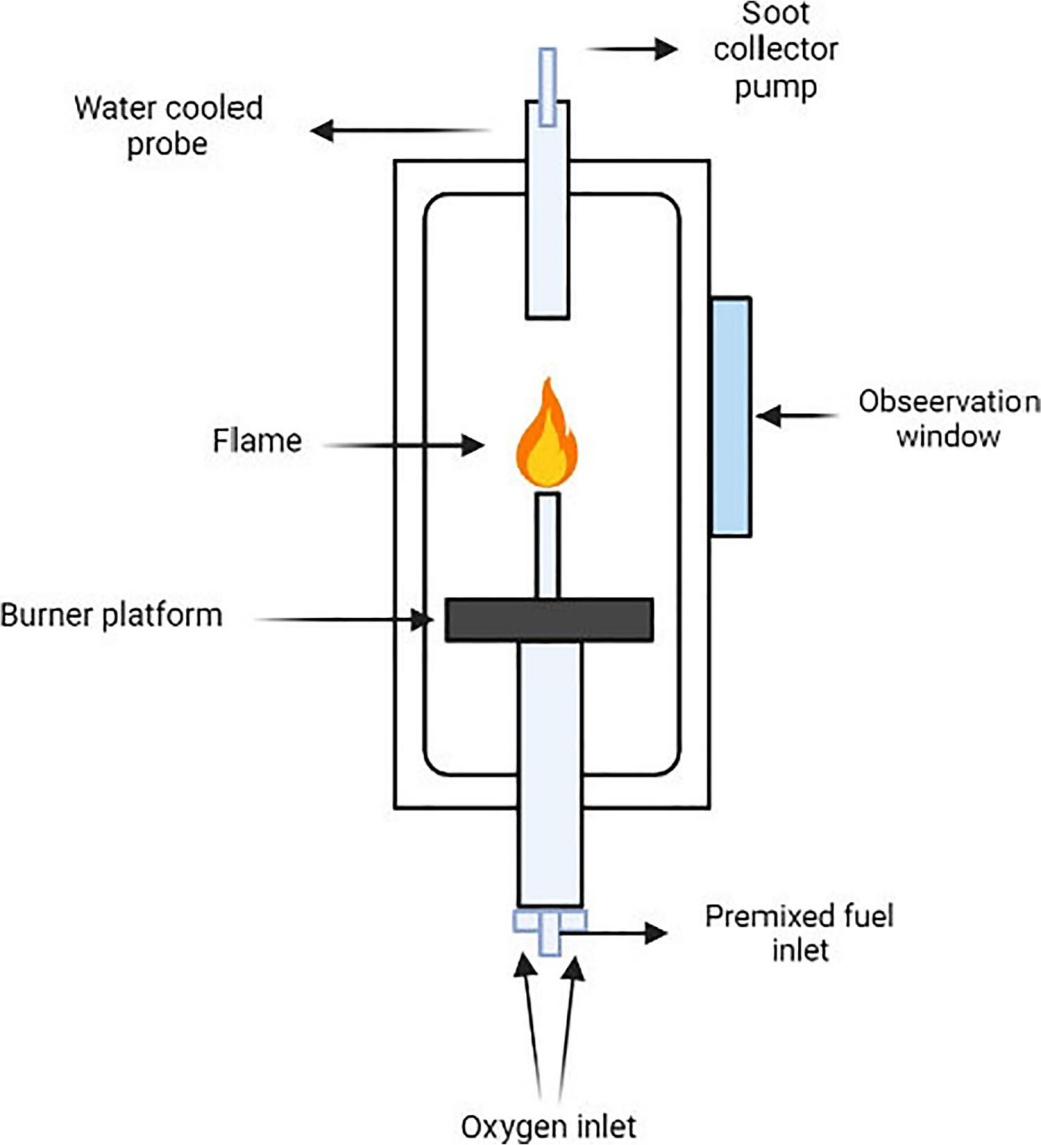
Combustion method

The combustion of fuel occurs at the tip of the premixed flame and the soot formed is collected in the soot collector pump. The purification of the fullerene soot can be done in a similar manner to the arc-discharge and laser ablation method, i.e., by Soxhlet extraction (schematic shown in Fig. 4).

### Microwave method

In this technique, microwave energy provides the heat source for the fullerene production (Ikeda et al. 1995). The microwave energy helps to attain homogenous heating of the precursors.

#### From graphite powder

The graphite powder which serves as a carbon source is filled in a borosilicate/SiO_2_ microwave vial. Microwave energy with a frequency of 2.45 GHz is applied to the vial and after the process, the complete soot is produced which depends on the intensity of microwave energy and the amount of graphite powder available (Ikeda et al. 1995).

#### From chloroform

In this process, a 250 W microwave energy and argon at a flow rate of 120 mL/min are introduced into the quartz column reactor. The chloroform is fed at 0.15 g/min into the direct current plasma torch located on the top of the quartz column reactor. Under the controlled operating parameters, fullerene fabrication takes place that is collected in the chamber. The fullerenes are extracted using toluene and further purified by HPLC.

### Chemical vapor deposition (CVD)

In contrast to the high temperature involvement in arc discharge and laser ablation method, the fullerene production by CVD occurs at a lower temperature (Kleckley et al. 1998). It involves the catalytic-decomposition of hydrocarbon vapors over transition metals. Kleckley et al. in 1998 presented two methods for the synthesis of fullerenes, namely hot-filament CVD and microwave-enhanced CVD.

#### Hot filament CVD

The hot filament CVD chamber made out of stainless steel consists of a tungsten filament that is suspended vertically with the upper terminal fixed and lower terminal attached to a braided copper wire. The filament current ranges between 50 and 60 A and the temperature of the filament ranges from 2000 to 2200 °C. The pressure in the chamber is allowed between 30 and 100 Torr and the hydrocarbon source which undergoes decomposition by the catalyst and forms soot is methane (Kleckley et al. 1998).

#### Microwave filament CVD

In this method, a quartz tube reaction chamber contains an excitation source which is a 100-W, 2.45-GHz generator. Acetylene, hydrogen, and argon are used as the feed gases and the typical pressures range from 1 to 10 Torr. At different pressures, the plasma generation and the chemical vapor deposition process vary. It was observed that at pressures less than 10 Torr, the quartz tube contained a yellowish colored film, which on exposure to plasma for 30 min, changed to dark brown, which was further scratched from the surface and purified (Kleckley et al. 1998).

## Extraction of fullerenes

The end product formed from all the fullerene synthesis methods is soot, which needs to be separated by an appropriate process (Keypour et al. 2013). For large-scale extraction of fullerene C_60_, the Soxhlet extraction process and column chromatography can be suitably employed. Using a Soxhlet extractor, the fullerenes can be extracted using various solvents, although toluene and benzene are the most commonly used solvents. This extraction process depends on the temperature, diffusion, and adsorption of solvents in the pores of soot.

The carbon soot containing the fullerene is added to the Soxhlet extractor and is extracted initially by toluene, which is then evaporated by a rotary evaporator, followed by the extraction of the fullerene from the soot using different solvents such as benzenes, dimethylnaphthalenes (DMN), N-methyl-2-pyrrolidone (NMP), and 1-chloronaphthalene (CN). The fullerenes obtained are then analyzed by various analytical techniques — HPLC, UV–Vis, FT-IR, XRD, NMR, and so on.

The column chromatography method is used for the analysis and purification of fullerenes. Here, the stationary phase employed depends on the affinity of fullerene. Silica gel is the most commonly used stationary phase but due to its lack of ability to separate the compounds, it has been used in combination with alumina.

## Functionalization of fullerenes

The biomedical applications of the unique properties of the C_60_ fullerenes hit a roadblock due to their extreme insolubility and hydrophobicity, which is called for modification in the parent structure. Functionalized fullerenes are the fullerenes with attached side chains, which can successfully exhibit high efficiency in forming singlet oxygen, hydroxyl radicals, and superoxide anions, and are considered effective mediators of PDT (Huang et al. 2014). The functionalization process increases the complexes’ dipole moment, allowing improved solubility in polar solvents and increasing the stability of the formed structures (Ferreira et al. 2021). The fullerenes offer numerous opportunities for functionalization and allow the attachment of a wide array of hydrophilic and amphiphilic side chains or fused-ring structures to the spherical fullerene core. The C_60_ molecule inherently lacks good aqueous solubility, thus causing its aggregation, and is tackled using mainly two approaches:

(i) Complexation with solubilizing agents to partially conceal the hydrophobic surface of the fullerene (Diederich and Gómez-López 1999)
(ii) Covalent functionalization at the fullerene surface (Diederich and Thilgen 1996)

The former accomplishment is obtained by complexing C_60_ with cyclodextrins to disperse them in water (Ikeda et al. 2013). The oxygen-based functional groups, mainly hydroxy, are introduced onto the fullerene surface using strong acids at high temperatures (Chiang et al. 1992). Another alternative to introduce the hydroxy group is using a basic solution of excess NaOH in water mixed with a suspension of C_60_ in benzene, catalyzed in the presence of a small amount of tetrabutylammonium hydroxide (Li et al. 1993).

**Fig. 5:**
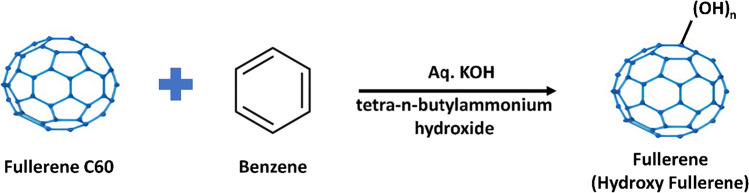
Formation of fullerenol

The fullerene C_60_-OH or fullerenol can be prepared using the following procedure:

Take C_60_ in a round bottom flask and dissolve it in benzene. Then, add aqueous solution of KOH and catalyst, tetra-n-butylammonium hydroxide slowly with stirring (shown in Fig. 5). Stir the reaction mixture at a room temperature until the color of the solution gets faded. Then remove the benzene using vacuum distillation and stir for another 60 h. Then, add deionized water to this mixture, stir it for 12 h, and dilute it again with water. Filter the mixture to remove the insoluble precipitates and give a wash with methanol to precipitate the C_60_–OH. Solubilize the obtained precipitate in water and separate on a Sephadex G-25 gel-filled size exclusion column. Freeze-dry the collected fractions to obtain C_60_–OH (Bai et al. 2021).

**Fig. 6:**
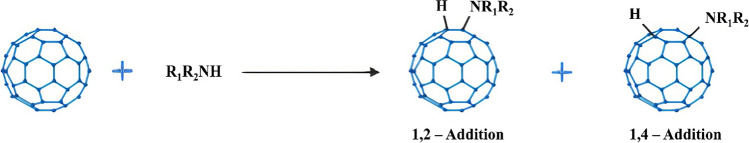
Formation of 1,2 and 1,4 amine substituted fullerene

Surface modification of C_60_ by grafting amine groups involves the mixing of fullerene C_60_ with different aliphatic primary amines (n-propylamine, t-butylamine, and dodecyl amine) (shown in Fig. 6). Some reactions also consider reacting C_60_ with smaller primary- and secondary-amine chains (methylamine, diethylamine). These amine-functionalized fullerenes are widely employed in gene transfection (Nakamura et al. 2000; Isobe et al. 2001).

Cyclopropanation is another alternative process that involves bonding an α-halo ester/ketone to the fullerene under highly basic conditions (Bingel 1993). The strong alkaline environment causes deprotonation of the 2-diethyl bromomalonate or methyl 2-chloroacetoacetate and a nucleophilic attack at the [6,6] position of the fullerene (Biglova and Mustafin 2019). The cyclopropanation reaction yields a modified fullerene that has increased water solubility as well as surface binding properties (Table 2).

**Table 2:** Surface modified fullerenes for clinical applications

Sr. no	Drug/carriers	Method	Outcome	Reference
1	Dihydroartemisinin	Dihydroartemisinin functionalization on C_60_ using 1, 3- Dipolar cycloaddition of azomethine ylide	Functionalizing C_60_ with dihydroartemisinin improved the aqueous solubility. Furthermore, nonlinear optical properties are considerably improved	Fouejio et al. 2021
2	R-thiazolidinethione (R: propyl, butyl, hexyl, phenyl)	Thiolation of R-thiazolidinethione on C_60_ fullerene	Functionalizing C_60_with thiazolidinethione increases the dipole moment of the formed complexes allowing higher solubility in polar solvents and improves (improving) the stability of the formed structures	Ferreira et al. 2021
3	Dendrofullerene (DF-1)	Dendro[60]fullerene is obtained via nucleophilic cyclopropanation of C_60_ with a 2nd generation bis(polyamide)-malonate dendrimer and deprotection of the terminal t-butyl groups	Radioprotection	Brettreich and Hirsch 1998, Daroczi et al. 2006
4	Fullerene–paclitaxel	The asymmetrical malonate obtained by treating tert-butyl N-(3-hydroxypropyl)carbamate with ethylmalonyl chloride was used to begin the synthesis of the C_60_-paclitaxel conjugate. Bingel-Hirsh addition to C_60_, followed by amino group deprotection was carried out	Cancer therapy	Zakharian et al. 2005
5	Fullerene-doped liposomes or lipid-membrane–incorporated C_60_ fullerenes (LMIC)	Fullerene exchange method from a γ-cyclodextrin (γ-CD) cavity to vesicles	Photodynamic cancer therapy	Ikeda et al. 2013, Doi et al. 2008
6	Fullerene-liposomes	Amphiphilic liposomal malonylfullerene (ALMF) and phospholipids co-assemble and form bilayer vesicles	Antioxidant property increased loading capacity	Lens et al. 2008
7	Fullerene vesicles	A water-soluble fullerene vesicle is generated where the R-group is substituted with C_6_H_5_, a phenyl (penta-) substituted fullerene cyclopentadienide (Ph_5_C_60_ K or PhK) and attains hydrophobic capabilities	Oxidative stress reduction	Maeda et al. 2008
8	Gadofullerenes	Phase transfer catalyzed (catalyzed) hydroxylation of Gd@C_82_	MRI contrast agents	Bolskar et al. 2003, Tóth et al. 2005, Sitharaman et al. 2007
9	Hydrophilic or cationic fullerenes	Functionalization of fullerenes with monocationic and tricationic dimethylpyrrolidinium	Photodynamic cancer therapy	Mroz et al. 2007, Qu et al. 2008
10	Human serum albumin-fullerene Fullerene hexaadducts PEG-modified fullerene	Complexation of HSA-C_3_ isomer with tris-malonic acid [C_60_] fullerene Pyropheophorbide-*a*-fullerene hexakis adduct conjugated to a monoclonal antibody Rituximab Water-insoluble fullerene (C_60_) conjugated with poly(ethylene glycol) (PEG)	Photodynamic cancer therapy Photodynamic cancer therapy Photodynamic cancer therapy	Belgorodsky et al. 2005 Rancan et al. 2002 Tabata et al. 1997
11	Fullerene polyamine (tetraamino fullerene)	Aminofullerene; tetrapiperidinofullerne is synthesized from fullerene, piperazine, and molecular oxygen	Gene delivery, transfection	Nakamura et al. 2000, Isobe et al. 2006
12	Amino-fullerene adducts	Amino-C_60_ adducts were prepared and functionalized with octa-amino and dodeca-amino groups by Hirsch-Bingel reaction	Non-viral gene delivery	Sitharaman et al. 2008
13	C60–cisplatin nanocomplex	Formation of non-covalent, entropically driven nanocomplexes between Cisplatin and C_60_ fullerene in physiological solution (i.e., the adsorption of Cis in C_60_ fullerene clusters)	Act as drug delivery carriers, mixture does not influence genotoxic Cisplatin activity and reduces the factions of necrotic cells	Prylutska et al. 2017
14	C60-berberine nanocomplex	Non-covalent complexation of herbal alkaloid Berberine with C_60_ fullerene	Act as drug delivery carriers, inhibiting the proliferation of CCRF-CEM cells	Grebinyk et al. 2019

Functionalizing fullerenes using glucose leads to glycofullerenes, which have numerous biological applications. Their synthesis is possible via reactions with diazoniums, azides, the Prato reaction, and the Diels–Alder reaction (Zhou et al. 2016).

## Fullerenes to target specific cancer pathways

**Fig. 7:**
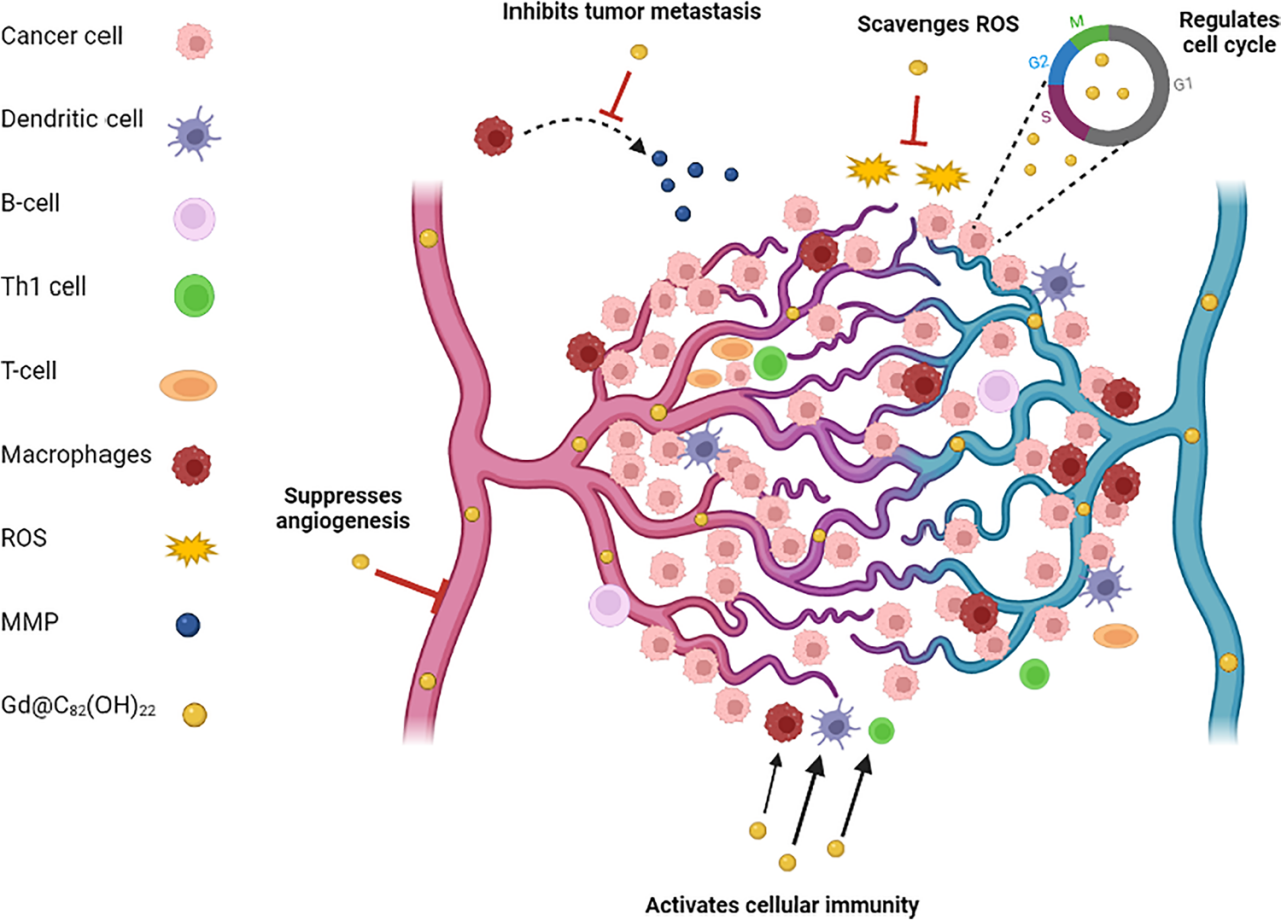
Depicts the activity of Gd@C_82_(OH)_22_ in cancer therapy acting as a metastasis inhibitor, ROS scavenger, cell cycle regulator, cellular immunity activator, and an angiogenesis suppressor

Fullerene mechanism through different pathways is given in Fig. 7 and Table 3.

**Table 3:** Surface functionalized fullerenes to target various cancer pathways

Primary molecule	Active therapeutic molecule	Role	Mechanism	Reference
Fullerene C-60/fullerene C-82	C_60_ (OH)_22_	Targets cancer stem cells	Inhibition of biological communication stem cells and tumor cells	(Nie et al. 2017)
	Gd@C_82_ (OH)_22_	Targets cancer stem cells	Reversal of phenotype of EMT in cancer cells	(Liu et al. 2015)
	Gd@C_82_ (OH)_22_	Inhibits angiogenesis	Downregulation of 10 proangiogenic factors in mice model	(Meng et al. 2010)
	C_60_ (OH)_20_	Inhibits angiogenesis	Downregulation of TNF-α, PDGF, and VEGF by 20–40% in EMT-6 tumor metastasis model	(Jiao et al. 2010)
	β-Alanine–Gd@C_82_ (OH)_22_	Inhibits Angiogenesis	Radiofrequency-mediated destruction of tumor vasculature	(Zhou et al. 2017)
	Gd@C_82_ (OH)_22_	Reactive oxygen species	Decrease in oxidative stress parameters in mice with H22 hepatoma	(Wang et al. 2006)
	Gd@C_82_ (OH)_22_, C_60_(OH)_22_	Reactive oxygen species	ROS scavenging capability as; Gd@ C82(OH)22 > C60(OH)22 > C60(C(COOH)2)2	(Yin et al. 2009)
	Gd@C_82_ (OH)_22_	Extracellular matrix	Decreased activity of MMP-2 and MMP-9 in tumor associated macrophages	(Meng et al. 2012)
	Iron oxide nanoparticles on PEGylated fullerene (C_60_)	Photodynamic therapy	Marked cytotoxicity in vitro and in vivo upon 532 nm light irradiation	(Shi et al. 2013)
	GO–fullerene hybrid	Photothermal and photodynamic therapy	High thermal ablation of cancer cells with increase in ROS production	(Li et al. 2017)

Nie et al. came up with C_60_(OH)_22_ nanoparticles, which were found to be highly efficacious in the treatment of cancer. It was known that proliferation and metastasis of tumors are promoted by the interactions between neoplastic cells and mesenchymal stem cells derived from the bone marrow. Also, it was found that 4T1 cancer cells of the breast could induce malignant differentiation of the stem cells which in turn influenced 4T1 cell growth and metastasis. Moreover, C_60_(OH)_22_ nanoparticles had the potential to block/attenuate the 4T1 cells and stem cell interactions thereby significantly suppressing tumor growth and metastasis. The MAPK signal activation in the stem cells suppressed metastasis. The pathways underlying were associated with a varied range of extracellular responses and were influenced by the secretion of various cytokines. The Erk- and p38-MAPK and its downstream NF-κB signal pathways of the malignantly differentiated stem cells were regulated by the C_60_(OH)_22_ nanoconjugates whereas in the case of normal stem cell regulation that occurred only via. Erk- and p38-MAPK and not by NF-κB activation (Nie et al. 2017).

Liu and colleagues devised a metal-fullerenol-based nanoparticle, Gd@C_82_(OH)_22_, which displayed an intrinsic inhibitory activity against triple-negative breast tumors while not causing any toxicity to normal epithelial cells of the mammary gland. It displays activity by causing the blockade of epithelial to mesenchymal transition, thereby eliminating cancer stem cells of the breast, subsequently curbing the initiation and metastasis of the tumor. Gd@C_82_(OH)_22_ mediates blockage of TGF-β signaling under normal oxygen conditions thereby preventing further tumor proliferation. Furthermore, conditions of hypoxia in the microenvironment of the tumor facilitate the nanoparticle uptake by the cells actively inhibiting the activities of HIF-1α and TGF-β, increasing the cancer stem cell eradication (Liu et al. 2015).

Another work by Meng et al. involved the development of a surface hydroxyl group functionalized Gd@C_82_(OH)_22_ nanoparticle, which at the mRNA level can simultaneously downregulate more than 10 angiogenic factors. In vivo studies performed for 2 weeks using Gd@C_82_(OH)_22_ nanocomposite displayed a more than 40% decrease in the tumor density in the microvessels and also a decreased blood supply to the tumor tissues (∼40%). The treatment efficacy was comparable to paclitaxel, without any prominent side effects (Meng et al. 2010).

A study by Jiao and team formulated C_60_(OH)_20_ fullerenol for cancer therapy, investigated using the EMT-6 metastasis model of breast cancer. The formulation was administered with 0.1 ml saline intraperitoneally in about 30 EMT-6 tumor-bearing mice and the effect was investigated. This novel molecule significantly modulated oxidative stress and also caused a reduction in angiogenesis factor expressions in the neoplastic tissues. A prominent decrease in the platelet endothelial cell adhesion molecule (CD31) expression and blood vessel density in the fullerenol-treated mice was observed in comparison with its controls which prevented the metastasis of the tumors in vivo (Jiao et al. 2010).

Zhou et al. devised a β-alanine functionalized gadofullerene nanoparticle which showed notable anti-neoplastic activity in H22 models of hepatoma. A 76.85% increase in the tumor inhibitory rate upon single treatment was observed owing to a strengthened radiofrequency (RF) interaction and extension in the time of blood circulation. This molecule acted as an anti-vascular therapy bringing about the physical destruction of the abnormal blood vessels of the tumor through the aid of RF. A long-term toxicity study of this nanoparticle in mice showed that it was eliminated after a few days with no significant organ toxicity, demonstrating its efficacy and biocompatibility (Zhou et al. 2017).

The potency of [Gd@C_82_(OH)_22_]_n_ nanocomposites to tackle oxidative stress was investigated by Wang and colleagues. It was observed that this molecule efficiently restored the kidneys and liver of the mice bearing tumors. Several enzymes, for instance, glutathione peroxidase, hepatic superoxide dismutase, catalase, and glutathione S-transferase, as well as the reduced levels of glutathione and malondialdehyde (MDA) protein-bound thiols, were compared between normal and tumor-bearing mice. It was observed that upon administration, there was a subsequent decrease in the oxidative stress-related enzymes and parameters, normalizing their levels. Furthermore, in vivo study performed proved that the nanoparticle could excellently regulate the reactive oxygen species production (Wang et al. 2006).

A study by Yin et al. proves that three water-soluble fullerenes such as C_60_(C(COOH)_2_)_2_, C_60_(OH)_22_, and Gd@C_82_(OH)_22_ can protect cells from oxidative damage caused due to H_2_O_2_ induction, stabilize the membrane potential of the mitochondria, and also decrease the production of ROS intracellularly in the order: Gd@C_82_(OH)_22_ ≥ C_60_(OH)_22_ > C_60_(C(COOH)_2_)_2_. These nanoparticles are found to cause the inhibition of in vitro lipid peroxidation and scavenge the stable 2,2-diphenyl-1-picrylhydrazyl radical (DPPH), and the reactive oxygen species (ROS) superoxide radical anion (O^2−^), singlet oxygen, and hydroxyl radical (HO) (Yin et al. 2009).

Meng and colleagues devised a metallofullerenol nanoparticle that potentially demonstrated anti-metastatic effects in invasive and metastatic models of breast cancer. The matrix metalloproteinase (MMP) enzyme production was blocked and interference with the cancer cell invasiveness in tissue culture conditions was observed. The treatment of the primary invasive tumor in the tissue invasion animal model with metallofullerenol nanoparticle showed a significantly lesser ectopic site metastasis with a decline in the MMP enzyme expression. Also, a fibrous cage formation was observed in the same animal model which encapsulated the cancer tissue and thereby hindered the communication between tumor- and cancer-associated macrophages terminating the production of metalloproteinase enzymes (Meng et al. 2012).

Shi et al. formulated an iron oxide nanoparticle–coated, PEGylated, fullerene C_60_ nanocomposite having enhanced biocompatibility and solubility and having superparamagnetic and powerful photodynamic properties. A novel photodynamic anti-neoplastic drug, hematoporphyrin monomethyl ether, was conjugated to the nanoparticle which exhibited a powerful photodynamic therapy. In contrast to the free drug, the drug-loaded carrier proved to be more efficacious in vitro on the cultured B16-F10 cells and in vivo on the murine tumor model portraying a 23-fold increase in the drug uptake in the tumor cells. Furthermore, the nanocomposite also acted as a T2-contrast agent for magnetic resonance imaging in vivo (Shi et al. 2013).

A novel GO-C_60_ hybrid containing methoxy polyethylene glycol and mono-substituted C_60_ was devised for combining photodynamic therapy and photothermal therapy using a stepwise conjugation method. The hybrid is soluble in a variety of environments, like physiological solutions. The addition of C_60_ to GO did not affect its photothermal properties but facilitated the generation of singlet oxygen (^1^O_2_) from C_60_ in the near-infrared region of an aqueous solution. This hybrid can also trigger reactive oxygen species production in Hela cells. Because of the synergistic effect of GO and C_60_, the GO-C_60_ hybrid outperforms both individuals in the cancer cell inhibition, indicating its high potential (Li et al. 2017).

## Factors affecting the activity of fullerenes

### Autooxidation

In presence of oxygen, the anionic fullerene radical can transfer one electron and produces a superoxide anion radical O_2_^−^ and hydroxyl radical OH (Yamakoshi et al. 2003; Bakry et al. 2007).

### Light and reducing agents

Few biological reducing agents such as reduced nitotinamide adenine dinucleotide (NADH), guanosin, and 6-thioguanine (6-TG) produce reactive oxygen species (ROS) with UVA irradiation or works as electron donor in some media. Quinones et al. (2014) demonstrated that C_60_ fullerenes in the presence of reducing agents and under visible light irradiation enhance the type-1 electron-transfer reactions and generate superoxide anion radical O_2_^−^. In the presence of UVA irradiation, C_60_ fullerenes and NADH undergo photochemical reactions to generate superoxide anion radicals which can further enhance the toxicity (Quinones et al. 2014).

### Solvent polarity

The critical solvent polarity in terms of solvent dielectric constant is essential for C_60_ and C_70_ aggregations. A student by Nath et al. (2002) demonstrated the C_70_ aggregations require almost double critical solvent polarity (i.e., from 27 to 31) compared to the C_60_ aggregations (i.e., 12 to 14). This study concludes that the highest differences in the critical solvent polarity values for aggregation of C_60_ and C_70_ are due to the polarizability of these two fullerenes (Nath et al. 2002).

Furthermore, the mixing of two different colloidal dispersion of fullerenes in an organic solvent enhances their stability (Alargova et al. 2001). Alargova and his co-workers performed an experiment on colloidal dispersion of C_60_ and C_70_. They prepared colloidal dispersions of C_60_ and C_70_ by mixing a fullerene solution in a good solvent with poor polar organic solvent for fullerene. The study reported the long-term colloidal stability of the samples without any stabilizer with the narrow particle size distribution pattern (Alargova et al. 2001).

### pH, ionic strength, and dissolved organic matter

The pH, ionic strength, and dissolved organic matter also affect the aggregation of fullerene C_60_ nanoparticles (nC_60_). In a study (Yang et al. 2013b), researchers investigated the effect of pH, ionic strength, and dissolved organic matter on the aggregation of nC_60_ in waste water. Study portrayed that at a pH 3 or higher ionic strength (> 100 mM NaCl), the aggregates’ size increases to greater extend which is reaching to micron size immediately after 1 h whereas in filtered waste water, the size remains same till 24 h. The concentration of dissolved organic matter in water highly influenced the aggregation rate of nC_60_ even at almost same zeta potential. It indicates the steric stabilization of nC_60_ aggregation owing to the adsorption of dissolved organic matter on nC_60_ aggregates present in waste water, in addition to the electrostatic stabilization (Yang et al. 2013b).

Freixa et al. studied the effect of fullerenes on the toxicity of organic micro-contaminants (OMCs) to river biofilms. Carbon nanoparticles such as fullerene C_60_ are considered very good adsorbent of OMCs. Hence, the interaction between fullerene C_60_ and OMCs can modify their toxicity to river biofilms or waste water. Therefore, in this study, investigators exposed the river biofilms with C_60_ and three OMCs such as triclosan/diuron/venlafaxine. Their investigation depicted that fullerene C_60_ does not only bear a risk for river microorganisms; even their presence with OMCs can increase (triclosan) or decrease (diuron) the toxicity to river biofilms (Freixa et al. 2018).

## Fullerenes in the diagnosis and treatment of tumors

Since 1985, the scene of fullerene-focussed research is drastically changing and owing to its boundless research and rapid advancements in the field of nanocarriers, fullerenes and their derivatives are gaining momentum in biomedical and clinical therapy. After overcoming the major hurdle of solubility by various modifications involving the physicochemical properties, functionalization, and development of metallofullerenes, this class of nanoparticles became significantly useful in the diagnosis and treatment of cancer. The fullerenes and their functionalized derivatives hold a distinguished and far-reaching collection of clinical applications, of which drug delivery, targeted imaging, and gene therapy have gained notoriety.

### Diagnosis

The distinct property of intra-fullerene electron transfer from the encaged metal atoms to the fullerene cages has proved to be of value in MRI (Kato et al. 2003) and thus the fullerenes and their derivatives can substantially improve the performance of MRI for the early detection of cancer as a nanoparticle-based contrast agent (Mody et al. 2009). A C80-BioShuttle-conjugate was reported to exhibit higher proton relaxation and improved signal enhancement at very low gadolinium (Gd) concentrations compared to other imaging contrast agents (Braun et al. 2010). GMF (gadolinium metallofullerene) is also gaining traction as a potential magnetic resonance imaging (MRI) contrast agent with extremely high relaxivity (Li et al. 2015a). By using functionalized metallofullerene (f-Gd 3 N@C_80_) in an orthotopic xenograft brain tumor model, researchers created a multimodal nanoplatform and exhibited longitudinal tumor imaging, sustained intratumoral probe retention, biodistribution, and extended survival rates (Shultz et al. 2011).

### Treatment

Fullerene derivatives like C_60_(OH)_20_ have shown significant improvement in termination of cancer cells primarily by cellular immunity activation (Liu et al. 2009a). They also exhibit inhibition of tumor cell proliferation in the EMT-6 breast cancer metastasis model (Jiao et al. 2010). The Gd@C_82_(OH)_22_ derivative was reported to have reduced the density of tumor micro-vessels by suppressing the angiogenesis (Kang et al. 2012) and has even shown inhibition of oxidative stress in the hepatocells (Wang et al. 2006). Various studies have shown that this fullerene derivative also acts by cell cycle regulation arresting the G0 phase (Meng et al. 2013) and that they inhibit MMP-2 and MMP-9 with high anti-tumor activity (Meng et al. 2012).

The phototoxicity of the fullerene derivative Hexakisamino-C_60_ was investigated in a squamous skin cancer cell line (A431) utilizing the MTT test and propidium iodide staining (Serda et al. 2020). The modified C_60_ was administered through the jejunum in rats, and it exhibited characteristics of an efficient drug carrier, namely slow drug release, reduced cytotoxicity, increased water compatibility, and improved bioavailability (Venkatesan et al. 2005). A C_60_-maleic anhydride derivative was administered for the mice with bone tumors which revealed photosensitizing action for antitumor effect (Jiang and Li 2007). Photodynamic therapy mediated by C_60_ is a promising option for reviving drug-resistant leukemic L1210 cell lines that have been driven to mitochondrial apoptosis (Franskevych et al. 2017). The pyrrolidinium-C_60_ derivative is an extremely effective photosensitizer and has reportedly killed three types of mouse cancer cell lines of colon adenocarcinoma, J774 reticulum sarcoma, and LLC, even at low concentration by exposure to white light. Additionally, this nanoparticular derivative causes cell apoptosis in CT26 cells following illumination and this resultant phototoxicity has been concluded to be mediated by O_2_ and the superoxide ROS (Tegos et al. 2005). Li et al. developed two amino acid-based nanoparticles C_60_-phe and C_60_-gly and evaluated these for photosensitizing activity for the liver cancer cell treatment (Li et al. 2014).

In mice, Gadofullerene NPs have been reported to have high anticancer effectiveness (60%) (Chen et al. 2005). They have been observed to have anti-angiogenetic characteristics and least toxicity with minimal side effect compared to the conventional treatment and thus may be a useful therapeutic strategy against angiogenesis in cancer therapy (Meng et al. 2010). The number of tumor micro-vessels was reported to have considerably decreased in fullerenol NP-treated tissues, which has been linked to lowering of the vascular endothelial growth factor (VEGF) expression (Jiao et al. 2010). In hepatoma models, β-alanine functionalized gadofullerenes have been shown to directly target tumor vasculature and due to the alteration with β-alanine, radiofrequency was employed as physical therapy after treatment with the nanocomposites, and the tumor inhibition rate was reported to be at 76.85% (Zhou et al. 2017). In 2011, Nakagawa et al. reported the cytotoxic/phototoxic, genotoxic, and mutagenic effects of fullerenols on HeLa, Chinese hamster ovary (CHO), human epidermal keratinocyte (HaCaT), and (human embryonic kidney) HEK293 cell lines respectively (Nakagawa et al. 2011). A newer class of fullerenes, the glycofullerenes, has been investigated as non-receptor tyrosine kinase inhibitors in the view of developing better nanotherapeutics for the treatment of pancreatic cancer (Serda et al. 2020). Inhibition of the S-phase cell cycle and induction of apoptosis can be achieved using a fullerene nanoconjugate in combination with gemcitabine (Nalepa et al. 2020). Prylutska et al. in 2019 reported that C_60_–cisplatin nanocomplexes increase the toxic effect of cisplatin on lung cancer cells in Lewis lung carcinoma cells (Prylutska et al. 2019). The C_60_-Berberiner nanocomplexes developed in a study were shown to inhibit the proliferation of CCRF-CEM (T lympoblastiod) cells (Grebinyk et al. 2019), while β-alanine functionalized gadofullerene nanoparticles possessed the ability to disrupt tumor vasculatures in melanoma cancer cells (Lu et al. 2020). Zakharian et al. developed a C_60_-paclitaxel conjugate as a slow-release medication for paclitaxel administration through aerosol liposomes for the treatment of lung cancer and studies revealed that the compound had anticancer activity comparable to free paclitaxel in vitro, and that it had the potential to improve the therapeutic effectiveness of paclitaxel in vivo (Zakharian et al. 2005). Another study on human epithelial lung adenocarcinoma A549 cells reported that the C_60_-paclitaxel conjugation showed tumor-suppressive action. Chaudhuri et al. in 2009 formulated Fullerenol-Dox nanocomplexes with minimal diameters (50–80 nm) and a drug-loading capacity of around 25% and reported that these effectively inhibit melanoma and Lewis lung carcinoma (LLC) cell growth and cause cell death in vitro, while in vivo, they prevent B16/F10 tumor development (Chaudhuri et al. 2009).

According to a recent study, the higher yields of photo-induced oxygen produced by β-alanine-modified gadofullerene nanoparticles (GFNPs) can result in the destruction of tumor vascular endothelial adherent junction protein-VE cadherin and reduction in tumor vascular endothelial cells-CD31 proteins, resulting in rapid tumor necrosis and proving to be a potent melanoma treatment (Lu et al. 2020). Fullerenes have also overcome chemotherapeutic drug resistance in tumors, and its derivatives have been utilized as antioxidant species (Kepinska et al. 2018). Funakoshi-Tago et al. in 2012 reported that by depleting apoptosis signal-regulating kinase-1 (ASK1) and inactivating the c-Jun N-terminal kinase (JNK) pathway, a water-soluble pyrrolidinium fullerene derivative, C(60)-bis (N,N-dimethylpyrrolidinium iodide), inhibits tumor cell growth and induces apoptotic cell death in cells mutated on JAK2 V617F, demonstrating a superior approach to treating myeloproliferative neoplasms (MPNs) (Funakoshi-Tago et al. 2012). [Gd@C_82_(OH)_22_] endohedral metallofullerenols have excellent antineoplastic effectiveness but low dosage and toxicity and require minimal drug loading to self-assemble into comparatively stable particles in a variety of solvents. They also have substantial antiproliferative effects on breast cancer cell lines MCF-7 (Chen et al. 2005). Sosnowska et al. used carbon nanostructures as an artificial extracellular matrix (ECM) and the results have shown that C_60_ nanofilms have the potential to be a substitute component for the ECM of cancer cells. The inclusion of fullerene C_60_ in the ECM may thus be an alternative therapeutic approach for hepatocellular carcinoma (Sosnowska et al. 2019). In another study, lysine-based C_60_-fullerene nanoconjugates for monomethyl fumarate (CF-LYS-TEG-MMF) delivery were synthesized and the proposed nanoconjugates had a pH-dependent drug release pattern, which reduced drug leaching at plasma pH. However, at cancer cell pH, the carrier provided the most drug release showing that internalization of drug molecules at the target location has a lot of potential for application in cancer therapy (Kumar et al. 2018).

## Toxicological aspects of fullerenes

The abundance of nanomaterials being developed for medical use brought their safety and toxicity following human exposure under scrutiny and a number of studies reveal that, although fullerenes have been proven to be extremely beneficial in various aspects, they do exhibit toxicity depending on several factors such as particle size, surface area, solubility, their biological accumulation, production of reactive oxygen species, lipid peroxidation, and method of preparation of fullerene dispersions in water. Hence, there is no standard method for the assessment of fullerene nanoparticle toxicity in aquatic environment (Ha 2014). Quick et al. performed a study to assess the effect of carboxyfullerene on the mice cognition and lifespan of mice. In this study, the mice with 12 M age were given C_3_ (e,e,e – C_60_(C(COOH_2_))_3_) at a dose of 10 mg/kg/day by dispersing C_3_ in drinking water. The study concludes that the C_3_ increases the survival rate of male and female mice and also enhances the learning and memory task (Quick et al. 2008). Usenko et al. (2007) evaluated the toxicity of fullerenes in embryonic zerbra-fish model and reported that the fullerene C_60_ at a dose of 200 µg/L significantly increases the malformations, pericardial edema, and mortality with necrotic and apoptotic cellular death. Jia et al. (2005) investigated the cytotoxicity of fullerene and reported the fullerenes at the dose of 226 µg/cm^2^ that showed insignificant cytotoxicity profile in alveolar macrophages. Nielsen et al. veraciously describes fullerenes and their functionalized derivatives as a “double-edged sword,” conferring upon them valuable features at low concentrations, but predicting inflammatory, cell-damaging, and carcinogenic properties at high concentrations (Nielsen et al. 2008). The pristine (underivatized) forms of fullerenes show much higher safety when compared to their derivatized forms (Nielsen et al. 2008; Trpkovic et al. 2012). Derivatizing or functionalizing the fullerenes significantly alters their chemistry, modifying their biological effects, including toxicity. Most pristine and functionalized fullerene preparations are not explicitly toxic, except when they are photoexcited or employed at higher concentrations (Shakirova et al. 2021). The penetration of fullerenes through the biological barriers, namely the blood-alveolus barrier, blood–brain barrier, and blood-placenta barrier, and their consequent accumulation in tissues can induce acute and long-term tissue injury. Since fullerenes have been reported to accumulate in various tissues, primarily in the liver, kidneys, lungs, and spleen, based on the exposure route, appropriate caution should be adopted to prevent their potential toxicity.

Initial studies conducted by Zhu et al. showed that non-functionalized fullerenes cause oxidative stress in the brain and result in the depletion of glutathione levels in young bass species (Zhu et al. 2006). Sayes et al. predicted that insoluble fullerenes will induce oxidative cell damage even at comparatively low concentrations and that the toxicity will diminish as the fullerene cage becomes more entirely derivatized and water soluble (Sayes et al. 2005).

Investigations revealed that while they cause no overt acute or chronic macro-toxicity, pristine and functionalized C_60_ presented superoxide-dependent and superoxide-independent genotoxicity in vitro, while low doses of pure C_60_ were shown to cause oxidative DNA damage in internal organs. This data necessitates vigilance concerning the potential of fullerene nanocarriers to escalate carcinogenic risk, hereditary disorders, and organ dysfunction even if it lacks apparent toxicity. Trpkovic et al. describe the toxicity of pristine and functionalized fullerenes, the mechanisms of cell damage, and delineate concentration, functionalization, preparation methods, solvent properties, and dimensional and surface topographical properties as the factors that chiefly impact the toxicity of fullerenes and the fullerene core. Inflammatory response, autophagy, apoptosis, DNA damage, and necrosis are some of the toxicological effects manifested by fullerenes (Trpkovic et al. 2012).

Prior to its application as a cytotoxic, an antioxidant, or a nanoparticulate drug delivery agent, the C_60_ molecule requires an extensive toxicity assessment. The various solubilization approaches utilized for C_60_ fullerenes (solvent exchange, mechanical processing, and long-term aqueous stirring) and fullerene functionalization processes determine its physicochemical characteristics and alter the superoxide-related activity and toxicity (Markovic and Trajkovic 2008). A study revealed that water soluble C_60_ fullerenes and hydroxylated fullerenes or fullerenes modified using cyclodextrin, surfactant, or polymer solutions generate photo-induced reactive oxygen species that bear phototoxicity towards keratinocytes (Zhao et al. 2008a, 2008b). They reported that the mechanisms of superoxide generation and cytotoxic activity may be a consequence of the porosity of the fullerene coating and the chemical properties of the solubilizing agent/ polymer/ surfactant used. Since pure and functionalized C_60_ fullerenes have access to intracellular space and possess the ability to accumulate at the cell membrane, they constrain cell functionality and stability (Foley et al. 2002; Sayes et al. 2005; Porter et al. 2006; Chirico et al. 2007; Li et al. 2008; Su et al. 2010; Zhang et al. 2011). Biological properties such as biodistribution, plasma protein binding, and cell uptake are affected by the surface charge of the nanocarriers (Lucafò et al. 2013). Research conducted by Bosi et al. showed that cationic fullerene derivatives with improved hydrophobic/hydrophilic surface ratio gain access to cells easily and comparatively show more toxicity than the neutral and anionic counterparts (Bosi et al. 2004), which indicates that increasing positive surface charge raises toxicity levels.

The approach of the fullerene core modification reduces its ability and the production of ROS (Prat et al. 1999; Bensasson et al. 2001) while improving the superoxide-quenching capacity, demonstrating that fullerene derivatives could have lower toxicity than their pristine forms (Sayes et al. 2004; Markovic and Trajkovic 2008).

The propensity of the C_60_ molecule to form aggregates is extensively dependent on the degree of functionalization of fullerene (Trpkovic et al. 2012). Consequently, monofunctionalized molecules display higher aggregation than polyfunctionalized fullerenes, which present with better stability profiles (Hotze et al. 2008). It has been reported that C_60_ functionalization substantially lowers the toxicity of fullerenes. Thus, the water-soluble fullerene derivatives manifest lower cytotoxic potential, presumably owing to their reduced ability to generate ROS, which results from the higher number of covalently bonded functional groups (Hamano et al. 1997; Prat et al. 1999; Bensasson et al. 2001; Sayes et al. 2004).

## Recent patents on hybrid nanostructures

There are a few patents filed in the area of fullerenes used for cancer treatment. They are listed in Table 4.

**Table 4:** List of fullerene patents for cancer therapy

SL. no	Patent no	Title	Description	Reference
1	JP2005053904A	Fullerene and anticancer therapeutic agent	The current invention pertains to an anticancer therapeutic drug for use in neutron capture treatment (NCT), which is widely anticipated in the medical industry due to cancer tissue’s specific therapeutic capabilities	(Kasama et al. 2005)
2	KR101479858B1	Fullerene nanogel prodrug for anticancer therapy	The current invention pertains to photodynamic treatment, namely the utilization of acid-activated fullerene nanogel prodrugs as cancer therapeutic agents and photosensitivity prodrugs. The current invention pertains to a new molecule for use in photodynamic treatment for malignant tumors that generates much better tumor selectivity and singlet oxygen than standard porphyrin-based photosensitizers, as well as a method for making it	(이은성 et al. 2015)
3	WO2007033578A1	Metallo-fullerenols and its application in preparation of medicines for inhibiting the growth of tumor	The innovation concerns a new nanomaterial and its use in biomedicine. It concerns a metal fullerene nanoparticle with the formula M@C 2 m (OH) × and its usage in the creation of a tumor-inhibiting medicament, where M is chosen from the group of rare earth metals such as Gd and La	(Zhao et al. 2007)
4	WO2018064963A1	Use of fullerene structure in preparation of medicament for treating tumor	The use of a fullerene structure containing at least one active ingredient selected from the group consisting of an oil-soluble hollow fullerene, an oil-soluble metallofullerene, a composition of the oil-soluble hollow fullerene and the oil-soluble metallofullerene, a combination of the water-soluble hollow fullerene and the water-soluble metallofullerene, a pharmaceutically acceptable ester of the water-soluble hollow fuller A pharmaceutical composition, a health care product, or a health food including the fullerene structure is also provided. A technique for producing the fullerene structure is also presented	(王春儒 et al. 2018a
5	CN107913289A	Application of the water-soluble fullerene structure in the medicine for preparing treatment tumor	The invention proposes a method of using a water-soluble fullerene structure in medicine to prepare therapy for tumors. The water-soluble fullerene structure contains the following: empty fullerene that is water-soluble and contains at least one pharmaceutically relevant salt or ester. The pharmaceutical is compatible with the human body, has minimal toxicity, is effective at suppressing tumors, and has a favorable therapy impact on tumors	王春儒 et al. (2018b)

## Conclusion and future prospective

Cancer nanotechnology has adeptly yielded the potential to provide innovative approaches for early cancer detection, leading to improved diagnosis and therapy. The traditional imaging approaches are highly intrusive, non-specific, and typically associated with tumor and solid cell damage. In recent years, as advances in commercial manufacturing of fullerenes and their functionalized derivatives have been realized, the research in fullerene application has also made significant progress. Fullerene derivatives have been demonstrated in studies to exhibit a wide range of anti-tumor properties, including immunological boosting, anti-oxidation, anti-metastasis, cell cycle arrest, tumor angiogenesis suppression, and multi-drug resistance inhibition. Nonetheless, fullerene derivatives must solve several critical challenges to overcome the limits of existing anti-tumor medicines and an emphasis needs to be laid on the effective control mechanisms of tumor growth and metastasis.

Currently, all fullerene research is limited to in vitro and in vivo preclinical studies and has not yet leaped into the stage of clinical trials. Prior to utilization as a drug carrier and therapeutic agent, extensive research is necessary to investigate the routes taken by the carbon nanocarriers to reach the target tumor tissues, their drug release mechanisms, and their potential for direct cancer therapeutics. Furthermore, there is inconsistent information on the toxicity profiles of these carbon nanocarriers on various animal models, their biological safety profiles, and metabolic processes in the body. These significant considerations bear an impact on the effective clinical use of the fullerene derivatives.

Since fullerene derivatives have distinct and intriguing optical, electrical, and magnetic characteristics that are primarily reliant on the methods used to manufacture and purify them, comprehensive knowledge about the property-determining characteristics and meticulous syntheses with regulated morphology and size are required to deliver the desired functionalities. Additionally, there is no defined characterization methods for nanocarriers and because their biodistribution and protein interactions are mainly surface- and size-dependent; they are expected to disperse differentially in a diverse sample and result in undesirable side effects or tissue toxicity, and hence, research in these areas must be a huge priority. However, with the vast technological advancements in every field and the impetus for innovation, the sphere of nanomedicine is assured to change with extensive therapeutic utilization of the fullerene nanoparticles.

## Data Availability

This is a review article, and hence, it is based on the earlier research in the proposed area.
